# Supplementary material for: Steady expression of high oleic acid in peanut bred by marker-assisted backcrossing for fatty acid desaturase mutant alleles and its effect on seed germination along with other seedling traits
Source: PLoS One. 2019 Dec 12;14(12):e0226252. doi: 10.1371/journal.pone.0226252 (PMC6910123; doi:10.1371/journal.pone.0226252)
Supplement: S2 Table — (DOCX) [file pone.0226252.s002.docx]

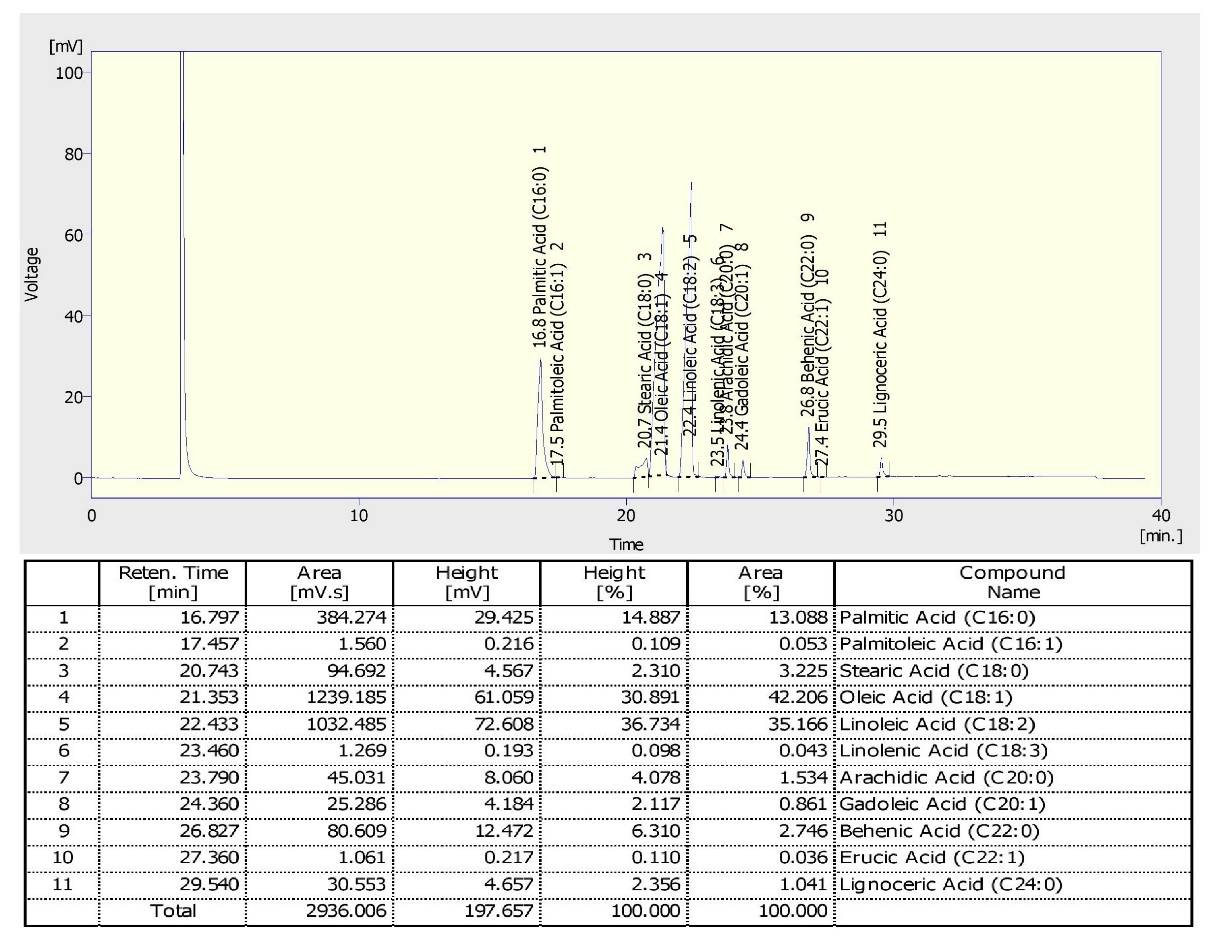


Fatty acid profile of ICGV06100 planted in ICAR-DGR during 2014 post rainy season


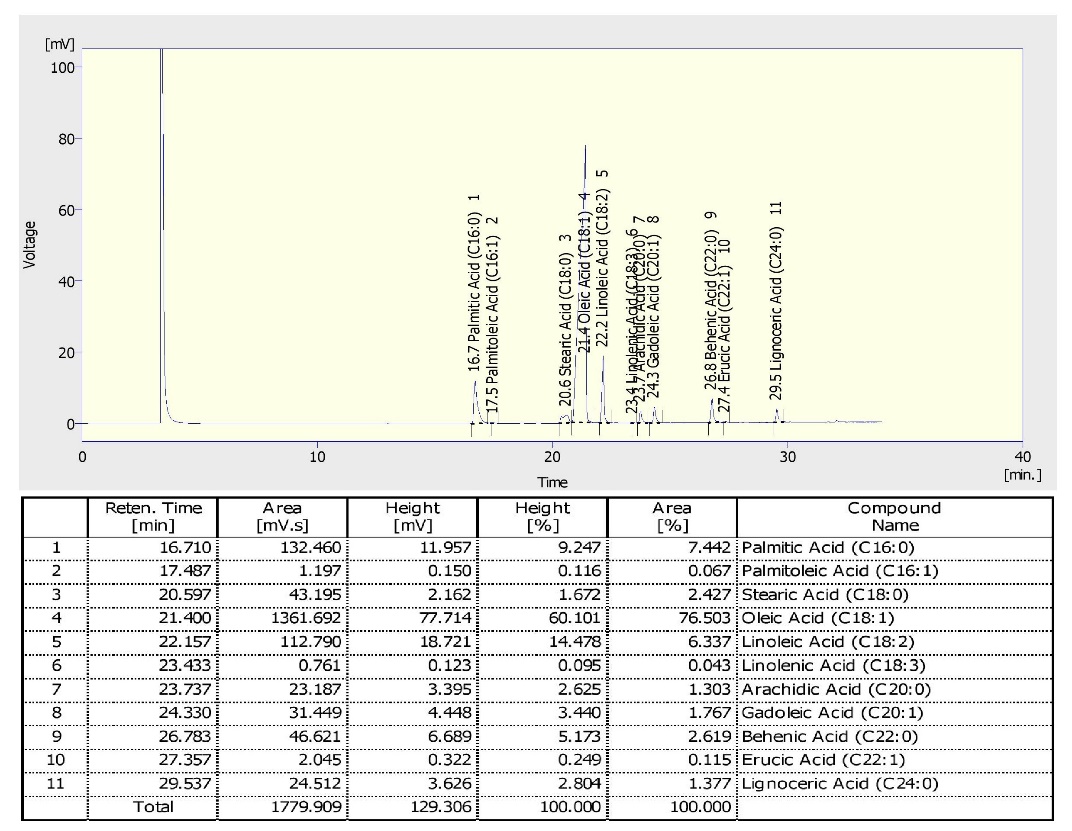


Fatty acid profile of SunOleic95R planted in ICAR-DGR during 2014 post rainy season


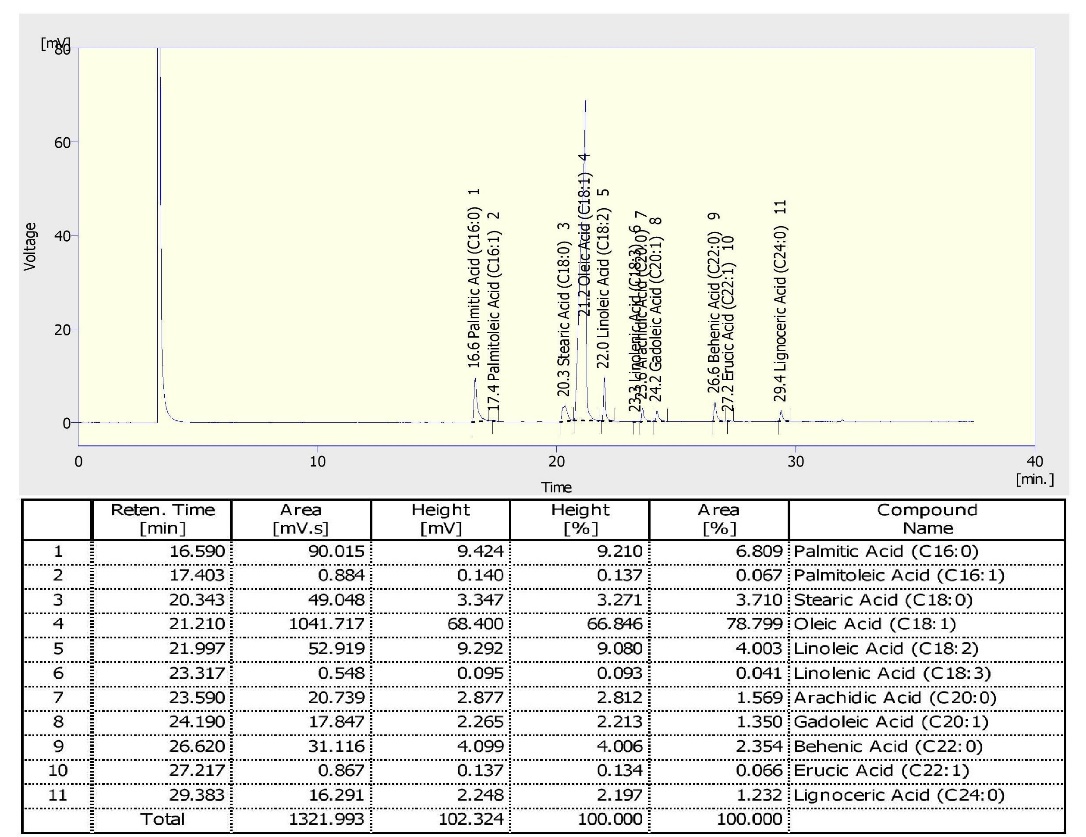


Fatty acid profile of NRCGCS-587 planted in ICAR-DGR during 2014 post rainy season


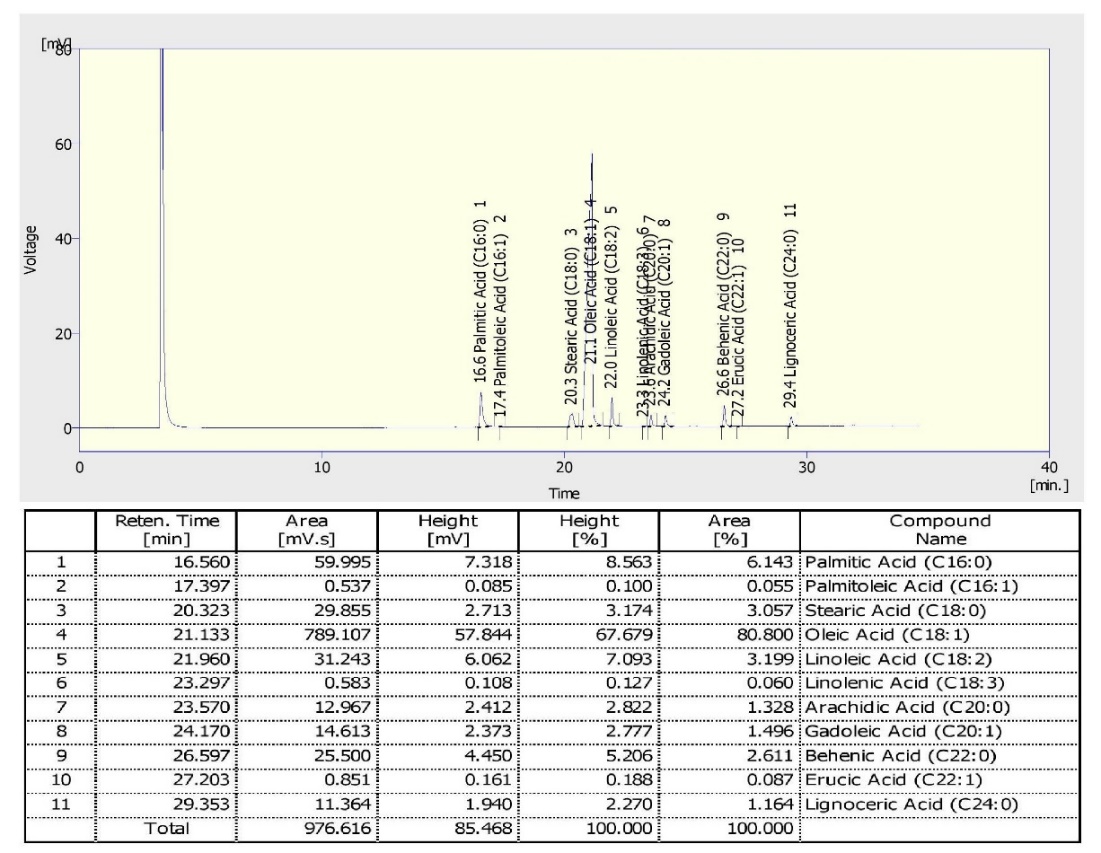


Fatty acid profile of NRCGCS-587 planted in ICAR-DGR during 2015 rainy season


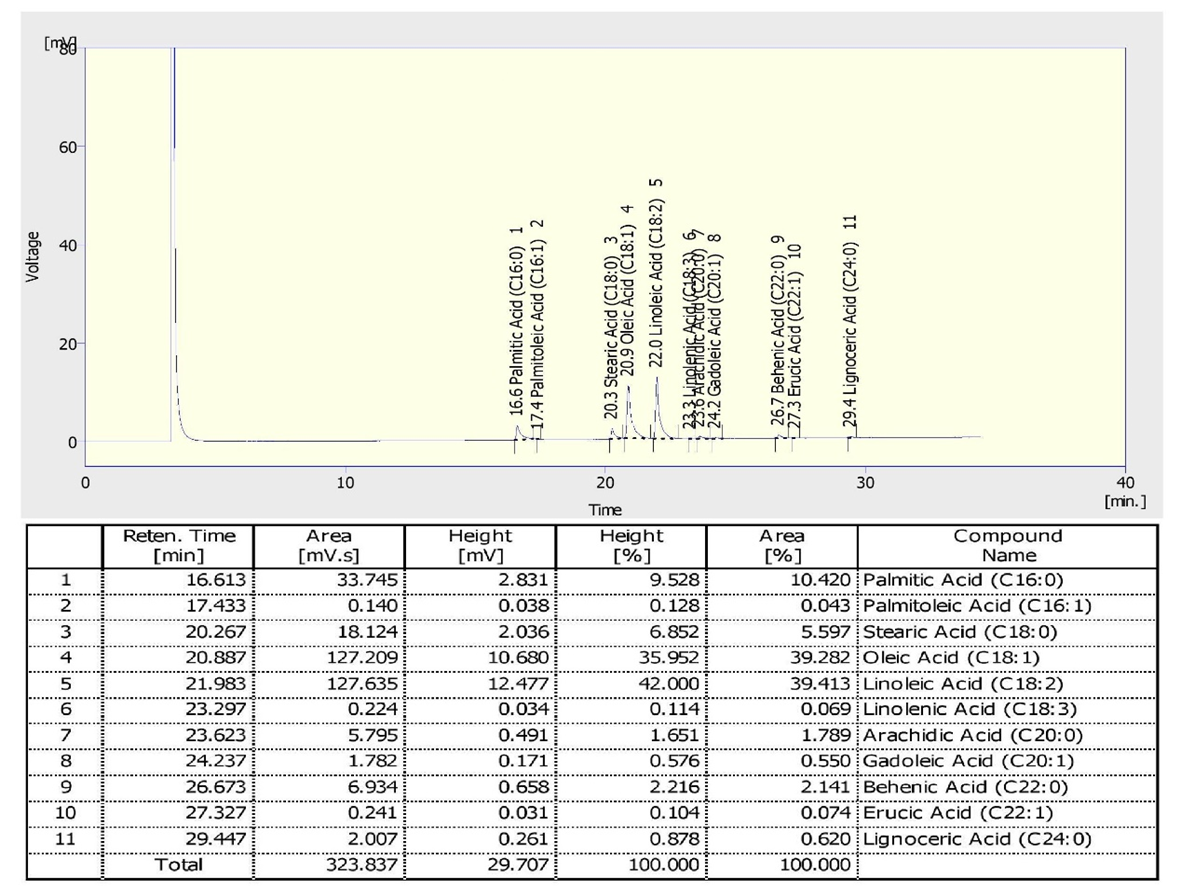


Fatty acid profile of ICGV06100 planted in ICAR-DGR during 2016 rainy season


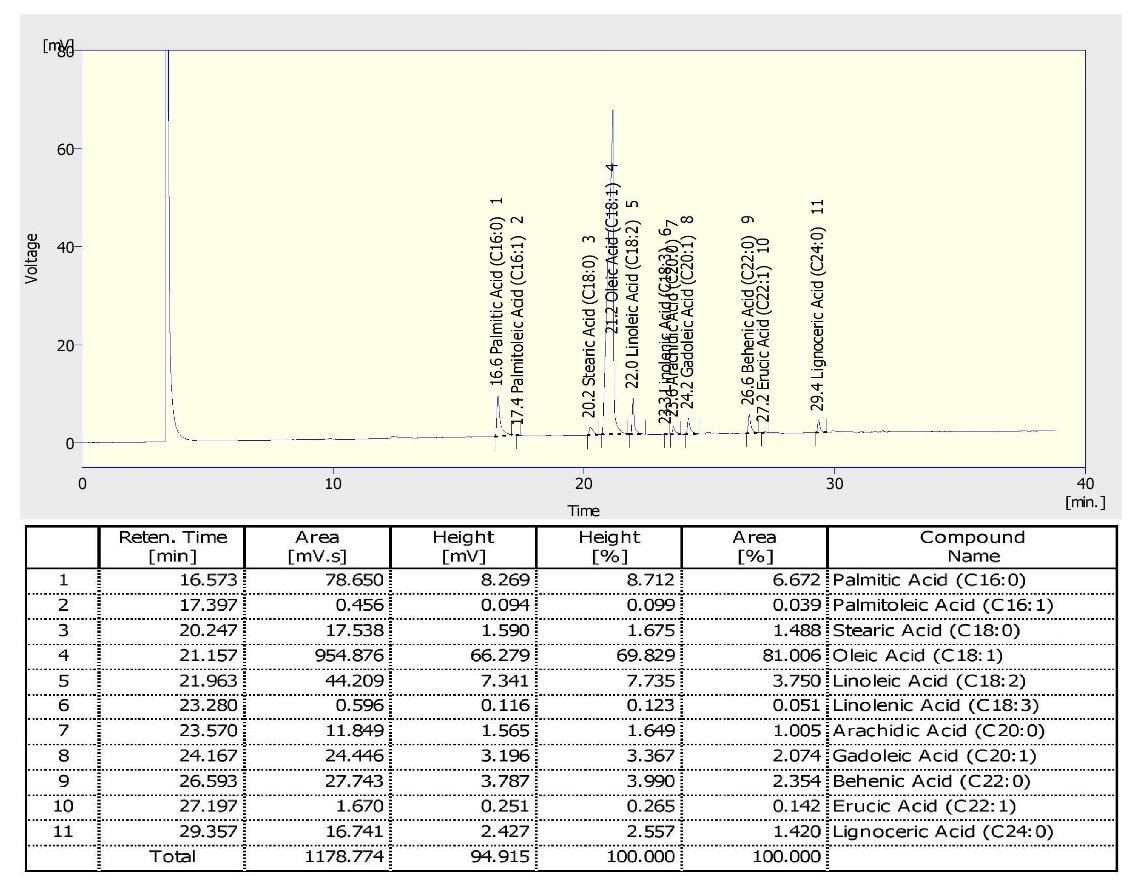


Fatty acid profile of SunOleic95R planted in ICAR-DGR during 2016 rainy season


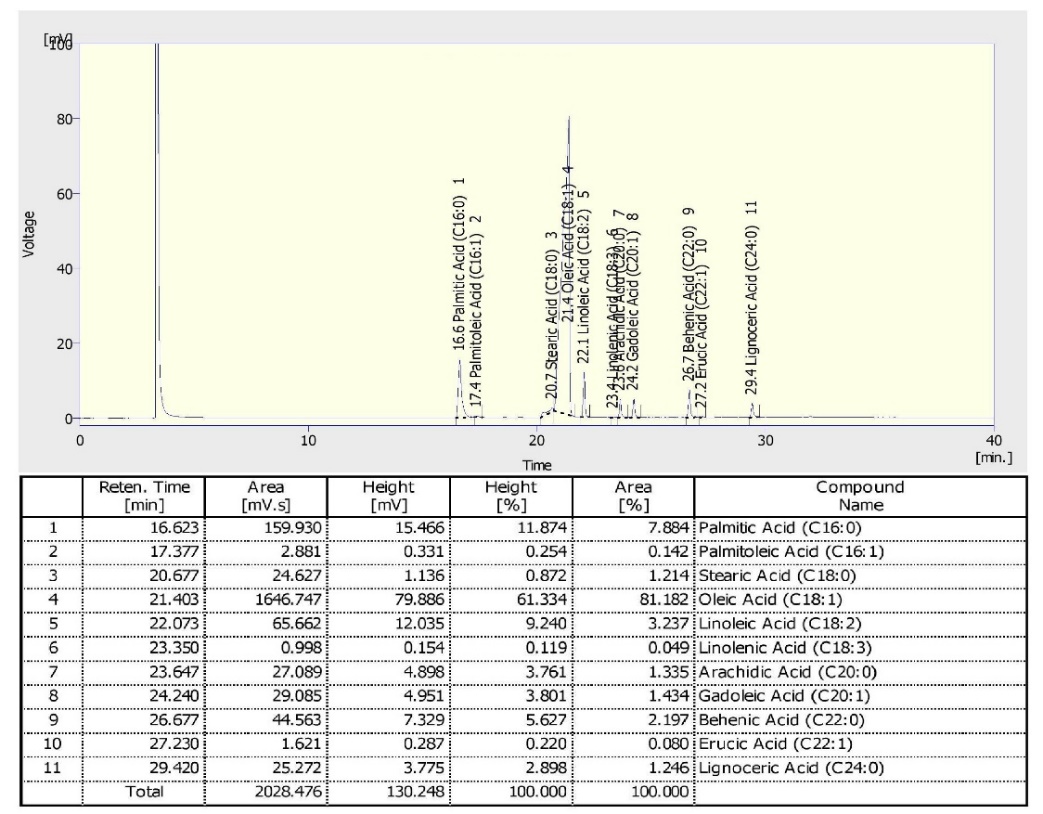


Fatty acid profile of NRCGCS-587 grown in ICAR-DGR, Junagadh during 2016 rainy season


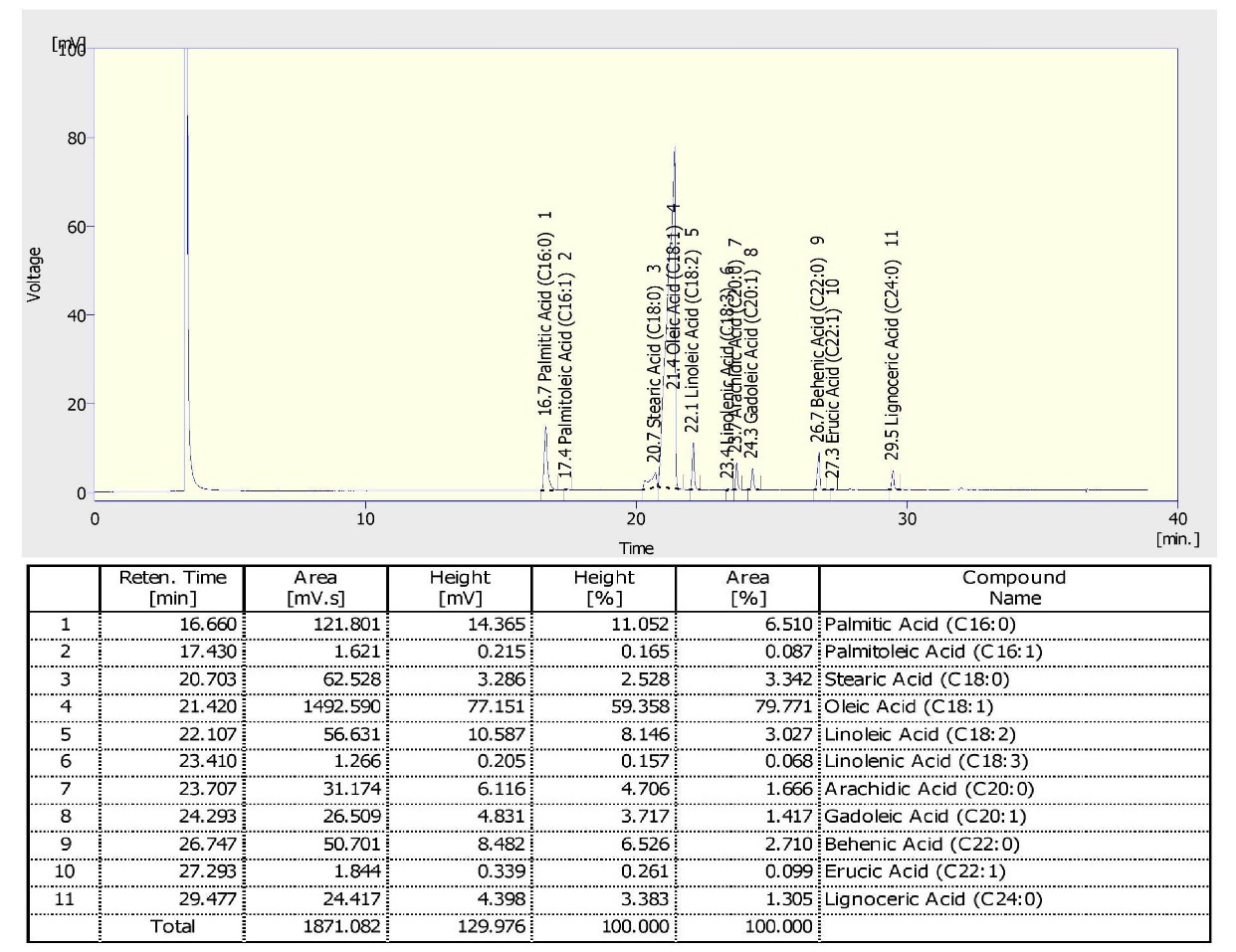


Fatty acid profile of NRCGCS-587 grown in ICRISAT, Telegana during 2016 rainy season


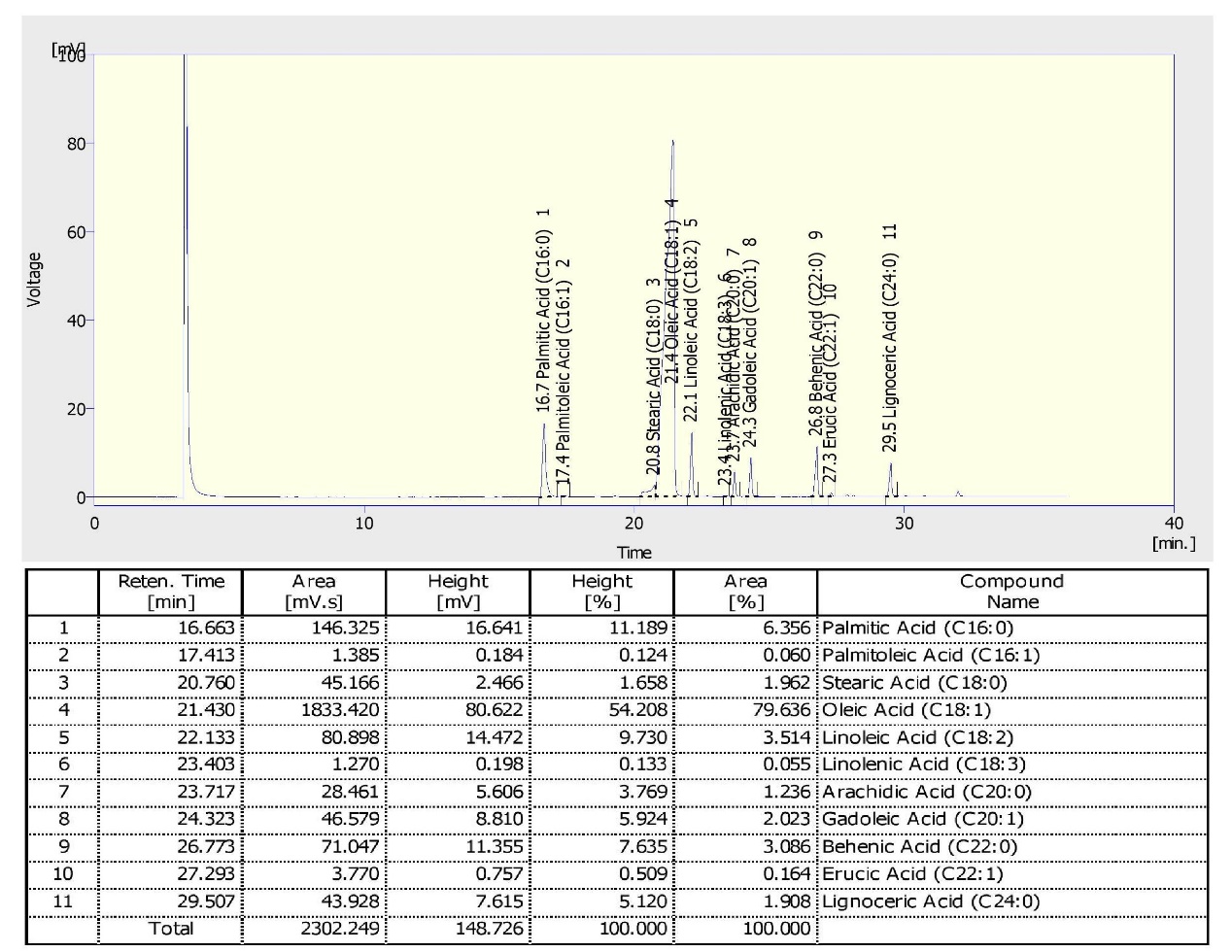


Fatty acid profile of NRCGCS-587 grown in RARS, ANGRAU, Tirupati, Andhra Pradesh during 2016 rainy season
