# Supplementary material for: Steady expression of high oleic acid in peanut bred by marker-assisted backcrossing for fatty acid desaturase mutant alleles and its effect on seed germination along with other seedling traits
Source: PLoS One. 2019 Dec 12;14(12):e0226252. doi: 10.1371/journal.pone.0226252 (PMC6910123; doi:10.1371/journal.pone.0226252)
Supplement: S3 Table — (DOCX) [file pone.0226252.s003.docx]

Qualitative, quantitative and special features of ICGV 06100 and NRCGCS-587 as per peanut descriptor

| **Qualitative traits** | |  | **Quantitative traits** | |  |
| --- | --- | --- | --- | --- | --- |
|  | NRCGCS-587 | ICGV 06100 |  | NRCGCS-587 | ICGV 06100 |
| Growth habit | 5-Decumbert-3 | Decumbert-3 | Days to 50 % flowering | 23 days after  germination | 25 days after germination |
| Branching pattern | Alternate | Alternate | Days to maturity | 115 | 120 |
| Stem hairiness | Nil | Nil | Plant height (cm) | 42.6 | 36 |
| Stem pigmentation | Light green | Green | Number of branches | 5 | 5 |
| Leaf colour | Green | Dark green | Number of flowers/ inflorescence | 2-3 | 2-3 |
| Leaflet shape | Ovate | Ovate | Leaflet length (mm) | 40.1 | 40.0 |
| Hairiness on young Leaflets | Yes | Nil | Leaflet width (mm) | 13.2 | 17.0 |
| Hairiness on mature Leaflets | No | Nil | Length / width ratio | 4:1 | 2:3 |
| Inflorescence type | Simple | Simple | Number of seeds per pod | 1-2 | 1-2 |
| Peg colour | Light green | Nil | Pod length (mm) | 26 | 27.6 |
| Standard petal colour | Yellow | Yellow | Pod width (mm) | 12.4 | 12.0 |
| Pod beak | Slight | Yes | Seed length (mm) | 13.8 | 13.3 |
| Pod constriction | Slight | Absent | Seed width (mm) | 6.8 | 6.6 |
| Pod reticulation | Slight | Yes | 100-seed weight (g) | 42.00 | 41.00 |
| Seed colour | Rose | Rose | Shelling percent | 72 % | 71% |
| Secondary seed colour | Nil | Nil | Pod yield (g/m^2^) | 221.00 | 206.00 |
|  | |  | Harvest index (%) (Pod basis) | 26.00 | 25.00 |
| **Special features** | | | | | |
| Reaction to Rust | 2 | 2 | Oil (%) | 55 | 54 |
| Reaction to late leaf spot | 3 | 3 | Oleic acid (%) | 80 | 39.28 |
|  |  |  | Linoleic acid (%) | 4 | 39.41 |
