# Supplementary material for: Steady expression of high oleic acid in peanut bred by marker-assisted backcrossing for fatty acid desaturase mutant alleles and its effect on seed germination along with other seedling traits
Source: PLoS One. 2019 Dec 12;14(12):e0226252. doi: 10.1371/journal.pone.0226252 (PMC6910123; doi:10.1371/journal.pone.0226252)
Supplement: Attachment — Submitted filename: Response to Reviewers.docx [file pone.0226252.s004.docx]

| **Reviewer** | **Comments** | **Reply** |
| --- | --- | --- |
| Reviewer 1 | **Comment 1:** The authors tried to describe a whole process for development of high oil and high oleate cultivars. The experiment was not specifically designed for any genetic studies or chemical analysis | **Reply 1:** We accept the comment partially.  Main aim of the experiment was to develop the introgression lines for high oleic traits which we have been developed successfully.  Once we developed the introgression lines then only we aimed for genetics studies and analysis of biochemical parameters and effect of high oleic acid on seed traits which are in sequence one after another. Hope This properly justify our experimentation. |
|  | **Comment 2:** In the title, steady expression of high oleic acid and its effect on seed germination along with other seedling traits did not match the manuscript content. The evaluation of these traits was the selection outcome and did not necessary related to high oleic acid. | **Reply 2:** We beg to differ from the comment of reviewer. The title of the manuscript clearly describe three components of studies conducted by us.  Component 1: Steady expression of high oleic acid in peanut  Component 2: (high oleic peanut) bred by marker-assisted backcrossing  Component 3: (High oleic acid ) its effect on seed germination along with other seedling traits  Hence, our manuscript exclusively describe the above three components and their results. Hence, the title clearly match the manuscript. |
|  | **Comment 3:**  **Point-1:** Even before making the cross, authors did not genotype parent ICGV 06100 for *FAD2* genotype.  **Point-2:** On Figure 2a and b, it was so confused. The well images were so different, but they scored them into the same genotype (for example, on Figure 2a well 6 and 7 to Aa; Figure 2b well 5, 6, and 7 images are the same but scored to different genotype Bb, BB, Bb). In addition, Figure 1a, only 4 samples but the authors mentioned 1 to 5. Figure 1b image was not clear.  **Point 3:** Furthermore, in the introduction (lines 79-81), the authors mistook gadoleic acid (C20:1) as saturated fatty acid. | **Reply 3:** We beg to differ from the comment of reviewer.  **Point-1:** Genotyping of ICGV 06100 was very much included in the gel picture (Figure 2a and 2b). In the figure 2a and 2b lane marked as P1 depicts the recurrent parent-1 which is ICGV 06100. However, we regret to mention here that labelling in the figure 2a and 2b was not proper which we have corrected subsequently.  **Point-2:** We wholly accept the comments of reviewer for mismatching the labelling and quality of Fig. 1a, 1b 2a and 2b.  Fig (1a, 1b, 2a and 2b) have been replaced by clear gel pictures with proper and corrected labelling. Also scoring details of these gel pictures have been added in Materials and methods (DNA extraction and marker genotyping subheading) to make the scoring clear.  **Point 3:** Necessary correction has been made in the manuscript. |
|  |  |  |
| **Reviewer 2** | **Comments** 1: Table 1 results are not included in the results section | **Reply 1:** We accept the comment of reviewer.  We unknowingly made mistake to mention table 1 in the manuscript which we have included under subheading “Recurrent parent genome recovery and linkage drag” of result section**.** |
|  | **Comment 2:**  Line 274 to 282 is this data represented in one of the figures or tables? | **Reply 2:** We accept the comment of reviewer. We have made necessary correction and mentioned “Table 1” in the “Recurrent parent genome recovery and linkage drag” of result section |
|  | **Comment 3:** Line 291 what is the p-values for Figure 3? | **Reply 3:** We accept the comment of reviewer. Necessary correction has been included as P value at 5 % level of significance (added in footnote of Fig.3) |
|  | **Comment 4:** Line 294 p-value?, Line 296 25% protein contents, should state "with no significant differences??"" and cite the p-value associated | **Reply 4:** We accept the comment of reviewer. Necessary correction (one sentence of no significant difference as depicted in figure 4 has been added in manuscript). P value at 5 % level of significance (added in footnote of Fig.4) |
|  | **Comment 5:** Line 298 p-value | **Reply 5:** We accept the comment of reviewer. p-value is at 5 % level of significance (added in footnote in Fig.3) |
|  | **Comment 6:** line 305 pod yield, p-value???? | **Reply 6:** We accept the comment of reviewer. p-value at 5 % level of significance added in the table 1. |
|  | **Comment 7:** Line 311 shelling percentage in table 1 data, what are the stats and p-value?? | **Reply 7:** We accept the comment of reviewer. We have used Duncan’s Multiple Range Test (DMRT) for test of significance at 5%. |
|  | **Comment 8:** Line 314 what are the p-values for the significant interaction effects?? | **Reply 8:** We accept the comment of reviewer. Significance at 5% included in table 2. |
|  | **Comment 9:** Line 321 where is this data table??? Line 323 p-value is needed?? Line 328 p-value is needed to support statement. | **Reply 9:** We accept the comment of reviewer. Results described in the results section is completely based on figure 6 and figure 7. We depicted the data in bar diagram only not mentioned in a fresh table. The numerical values of oil content, Oleic, Linoleic and palmitic acids between states were more or less similar. Hence neither we compared it statistically nor added as a separate table. Hence, no p-value is mentioned. |
|  | **Comments 10:** Line 351 mention significant differences if any her in detail and in table 3, state p-value | **Reply 10:** We accept the comment of reviewer. P-vale at 5% included in the Table 3. |
|  | **Comment 11:** Line 361 to 362 please re-word for clarity is confusing as written | **Reply 11:** We accept the comment of reviewer. We have edited the sentence to make to make meaning clear |
|  | **Comment 12:** Table 1, table footnotes are needed, define importance of superscripts statistically, what are the p-values | **Reply 12:** We accept the comment of reviewer. We have made necessary correction in the Table 1. |
|  | **Comment 13:** Table 2 define in table footnote how yield mean sq was determined, describe in footnotes the 3 locations, define PC1, etc, briefly state in footnotes stats used | **Reply 13:** We accept the comment of reviewer. We have made necessary correction in the Table 2. Name of three locations were mentioned in the write up, hence avoid in the table to avoid clumsiness. |
|  | **Comment 14:** Table 3. table footnotes, table should in p-values, also, briefly state the methods used, stats used, so that reader can more clearly understand the data | **Reply 14:** We accept the comment of reviewer. Footnotes added in the table 3. Methodology and stats used have been mentioned in the material methods section. |
|  | **Comment 15:** Figure 1 and Figure 2 are never discussed in the results section. also if these figures are to be used they need to include figure legends with details of the methods and quantity of the DNA starting materials for amplification | **Reply 15:** We accept the comment of reviewer. Results of Figure 2 was already discussed in the manuscript. However, results of Figure 1 was missing. We have added the results of Figure 1(with legends) in Materials and methods (DNA extraction and marker genotyping), methods of genotyping has also been added and references have been added. |
|  | **Comment 16:** Figure 3, Figure 4, Figure 6. and Figure 7, figure legend is needed, p-values needed with standard error bards, bar graphs using the current colors is difficult to read, try black and white and different patterns, % of what?? % of total fatty acid, x-axis values?? | **Reply 16:** We accept the comment of reviewer. We have modified all the graphs accordingly. X- axis is self-explanatory (indicates % of total fat) |
|  | **Comment 17:** Figure 5???? not sure if this data adds to the strength of the manuscript?? | **Reply 17:** We accept the comment of reviewer.  Yes, Fig. 5 is needed as it indicates stability and high yield of NRCGCS-ILs over locations by GGE biplot analysis and to identify such genotype was ours second objective. |
|  | **Comment 18:** Figure 8 same as previous comments of Figure 3, 4, also what are the harvest times and from what three states, figure legend needed | **Reply 18:** We accept the comment of reviewer. Figure 8 is essential, as it shows the passport data of new identified line NRCGCS-587 (COMPARED WITH RECURRENT PARENT)  One sentence on harvest time i.e., Harvest on maturity of crop (110-115 days after sowing) has been added in Yield evaluation section of MABC-ILs in results section. |
|  | **Comment 19:** Figure 9. this figure is never discussed in the manuscript. either include and discuss in the results/discussion or remove. not sure if it adds to the strength of the manuscript. | **Reply 19:** We accept the comment of reviewer. Figure 9 has now been added in materials and methods part (seed and seedling traits), figure is a part of explanation of methodology followed to grow the seedlings to screen for important morphological traits to characterize the genotype. |
|  | **Comment 20:** **General comments** Line 71 and "the" rest Line 73 and the "remaining on-third" Line 74 low caloric (corrected spelling) line 105, remove "in a while" line 295 space after and line 297 space after and | **Reply 20:** We accept the comments of reviewer. We have incorporated all the corrections highlighted by the reviewer. |
