# Supplementary material for: Steady expression of high oleic acid in peanut bred by marker-assisted backcrossing for fatty acid desaturase mutant alleles and its effect on seed germination along with other seedling traits
Source: PLoS One. 2019 Dec 12;14(12):e0226252. doi: 10.1371/journal.pone.0226252 (PMC6910123; doi:10.1371/journal.pone.0226252)
Supplement: Attachment — Submitted filename: Response to Reviewers.docx [file pone.0226252.s005.docx]

Comments

| **Reviewer** | **Comments** | **Reply** |
| --- | --- | --- |
| #3: | The authors have responded to all questions raised by the previous reviewer except the question about the title. They need to change the title because it does not reflects the contents of manuscript. The title should mention the main achievement which is the development of high oleic content line. The title might read something like this "Development of stable lines with high oleic content through marker assisted introgression of fatty acid desaturate mutant alleles and its effect on seed germination". All other questions raised by previous reviewer has been answered and have improved the quality of manuscript significantly. | We beg to differ from the comment made by the reviewer #3. The reviewer #3 mentioned that the authors had not responded to the question about the change of title which is absolutely incorrect. During the first review reviewer #1 offered the same comment in his comment no.-2. In reply we submitted justification for not making any changes in the title of our manuscript (**Can be referred earlier review**). We still stand with our justification this time also and do not see any point for making changes in the current title of our manuscript. However, final decision lies with the editor. |
|  |  |  |
| #4: | I feel that the title may be modified. Currently, the title seems to indicate that variations in germination frequencies and other seedling traits are primarily due to high oleic acid, which may not be the case due to various reasons including genomic reconstitution as elaborated below. It would be advisable to list these as separate features/studies of the high oleic lines. | We beg to differ from the comment of the reviewer. The title of the manuscript clearly describe three components of the studies conducted by us.  Component 1: Steady expression of high oleic acid in peanut  Component 2: Breeding of high oleic peanut by marker-assisted backcrossing  Component 3: Effect of high oleic acid on seed germination along with other seedling traits  Hence, our manuscript clearly describe the above three components and their results. Hence, the title clearly match the manuscript. |
|  | In the abstract, the authors talk about resistance to biotic stresses but there is no mention of this data in the manuscript. This portion towards the end should be deleted | The data on resistance to biotic stresses (Rust and Late Leaf Spot diseases) of NRCGCS-587 and ICGV 06100 (recurrent parent) have been mentioned in supplementary Table–2 (S2). It may be treated as additional information. Hence, deleting the statement on resistance to biotic stresses from the text may not be required. |
|  | Line 66: "in around" | Accepted, Necessary correction has been made in the manuscript. |
|  | 1. Lines 191-195: Aren't four SSRs per linkage group too less for estimating recurrent genome reconstitution? | It is always better to use SSRs as many as possible for estimating recurrent genome reconstitution. However, we selected four anchored SSRs from each linkage group (altogether 80 SSRs) which are genetically mapped in the peanut consensus map. Out of which 79 SSRs gave similar expression both in recurrent parent and introgression line. It gives a fair indication about the reconstitution of the recurrent parent genome in introgression line. Furthermore, we used three cycles of backcrossing which essentially (mathematically) recovers 93.75% of the recurrent parent genome. Both these approaches are supplementary to each of above two approaches and confirm recurrent parent genome reconstitution. |
|  | 2. Why was backcrossing done only till BC3? Would it be sufficient to reconstitute a significant portion of the recurrent parent genome in the absence of background selection.  Since background selection was not done, in conjunction to point 1 above, there could be sufficient portions of the non-recurrent parent genome remaining in the resulting selected line which could be responsible for other variations observed in seedling traits. Also, this would raise questions about the stability of the generated line. | Theoretically, three cycle of backcrossing supposed to recover 93.75% recurrent parent genome. We attempted three cycles of backcrossing which helped us to recover 93.75% recurrent parent genome. In such case taking background selection additionally would not yield any more information. However, one can do any number of additional work to strengthen the result. Yes, there is a chance of having 6.25% of non-recurrent parent genome in the introgression line and literally, this 6.25% of non-recurrent parent genome may throw little or no variations among the introgression lines. |
|  | 3. Was stable inheritance of the high oleic phenotype tested using successive generations of harvested seed?  This is not clearly given in the text.  How was it done?  Which generations of selfed progeny were used? | Yes, we have tested the high oleic phenotype in F_3_, F_4_ and F_5_ generations and confirmed.  It is clearly mentioned in the text line no. 300 (for the year 2014), 308 (for the year 2015) and 337 (for the year 2016 in multiplication).  High oleic trait was phenotyped through fatty acid analysis of kernels in Gas chromatography (Details methodology have been described in Materials and Methods section).  Selfed progenies of F_3_, F_4_ and F_5_ generations |
|  | 4. How is passport data a good approach to test for genomic reconstitution? I am not in agreement with this approach. | Passport data of a genotype/verities of a particular crop is an important information which is used in DUS (Distinctness, Uniformity and Stability) testing. It helps to distinguish/identify one genotype from others. If two genotypes are similar in majority of the passport traits except one or two major trait(s), the genotypes can also be refereed as near isogenic line (NIL). Here, recurrent parent and introgression line are similar in majority of their passport traits except the high oleic content which we have introgressed into the introgression line. Theoretically, 3 cycle of backcrossing supposed to recover 93.75% genome of recurrent parent. Thus introgression line, NRCGCS-587 has got more than 90% genome constitution of recurrent parent (ICGV 06100), which has been reflected in the passport data of introgression line and recurrent parent. |
|  | Line 226: Shouldn't it be "111-115"? | Accepted, Necessary correction has been made in the manuscript. |
|  | Line 260: Any references for this formula? | Accepted, Necessary correction has been made in the manuscript. |
|  | Line 284: Should be "codified" or "named" or "termed" instead of "decoded" | Accepted, Necessary correction has been made in the manuscript. |
|  | Line 290: (RPG) in brackets | Accepted, Necessary correction has been made in the manuscript. |
|  | Line 292: What does "nine were amplified" imply?  Did they harbor the SunOleic95R genome segments?  Is there any information available on the genetic/physical distance of these 10 SSRs? | Accepted, Necessary correction has been made in the manuscript.  Yes, there is always possibilities, possible explanation is mentioned in the text.  Yes, Necessary information is already available in the supplementary table 1 (S1). |
|  | Line 368-369: "However, the..........the groups" is not clear. Rephrase. | Accepted, Necessary correction has been made in the manuscript. |
|  | Figure 1 legend: Please correct for language | Accepted, Necessary correction has been made in the manuscript. |
